# Factors influencing post-ICU psychological distress in family members of critically ill patients: a linear mixed-effects model

**DOI:** 10.1186/s13030-021-00206-1

**Published:** 2021-02-15

**Authors:** Rahel Naef, Stefanie von Felten, Jutta Ernst

**Affiliations:** 1 Centre of Clinical Nursing Science, University Hospital Zurich, Raemistrasse 100, 8091 Zurich, Switzerland; 2 Institute for Implementation Science in Health Care, Faculty of Medicine, University of Zurich, Zurich, Switzerland; 3 Department of Biostatistics, Institute of Epidemiology, Biostatistics, and Prevention, Faculty of Medicine, University of Zurich, Zurich, Switzerland

**Keywords:** Family, Intensive care, Anxiety, Depression, Posttraumatic stress, Satisfaction with care

## Abstract

**Background:**

Adverse responses to critical illness, such as symptoms of depression, anxiety or posttraumatic stress, are relatively common among family members. The role of risk factors, however, remains insufficiently understood, but may be important to target those family members most in need for support. We therefore examined the association of patient-, family member- and care-related factors with post-ICU psychological distress in family members in a general population of critical ill patients.

**Methods:**

We conducted a prospective, single-centre observational study in a twelve-bed surgical ICU in a 900-bed University Hospital in Switzerland. Participants were family members of patients treated in ICU who completed the Family Satisfaction in ICU-24 Survey, the Hospital Anxiety Depression Scale, Impact of Event Scale-Revised-6, and a demographic form within the first 3 months after their close other’s ICU stay. Data were analysed using linear mixed-effects models, with depression, anxiety, and posttraumatic stress as outcome measures.

**Results:**

A total of 214 family members (53% return rate) returned a completed questionnaire. We found that higher levels of satisfaction were significantly associated with lower levels of depression, anxiety and posttraumatic stress. There was no statistically significant association between family member characteristics and any measure of psychological distress. Among the included patient characteristics, younger patient age was associated with higher levels of depression, and patient death was associated with higher levels of depression and posttraumatic stress.

**Conclusions:**

Our results suggest that satisfaction with ICU care is strongly associated with family well-being post-ICU. Family members of younger patients and of those who die seem to be most at risk for psychological distress, requiring specific support, whereas family member characteristics may have less relevance.

## Background

Critical illness has a profound impact on the well-being of family members [1–3]. Families experience high levels of stress and uncertainty during a close other’s stay in an intensive care unit (ICU) [4–6], which negatively affects their mental health post-ICU [7, 8], particularly in the event of life-threatening illness that required surrogate decision-making and resulted in the death of the critically ill family member [8, 9]. Post-ICU psychological distress affects a considerable proportion of family members and includes symptoms of anxiety, depression, posttraumatic stress, complicated grief, and caregiver burden [10–12], which have been clustered under the term post-intensive care syndrome – family (PICS-F) [7, 13]. Prevalence of PICS-F in an adult population is reported to be between 20 to 60% within the first 3 months, but varies according to the specific symptom and ICU population studied, measures used, and the time-point of assessment [10, 13, 14]. Psychological distress not only increases family suffering, but affects family members’ functioning in everyday life, and limits their ability to engage in caregiving activities needed by the survivor of critical illness, who may also suffer from a adverse consequences in their physical and mental health [10, 14–17].

To reduce the prevalence of adverse family response to critical illness, attention to factors affecting post-ICU psychological distress is called for. A number or family member-, patient-, and care-related, potential risk factors have been put forward as influencing the incidence of psychological distress in these families [10, 11, 13–15]. Family member-related risk factors for post-ICU psychological distress include female gender, younger age, being a close relative, lower education, and prior mental health issues [8, 18–23]. Unexpected admission to ICU, length of stay, severity of illness, death and younger patient age have been defined to be patient-related risk factors for PICS-F [9, 10, 13, 19, 21]. In terms of care-related factors, satisfaction with ICU care [15, 24], poor communication that fails to meet needs or includes inconsistent information [8, 9, 11, 15], and involvement in end-of-life decision-making that does not correspond with families’ preferred decision-making style [9, 13, 19] have been found to be associated with adverse outcomes. While the literature consistently suggest that family-, patient-, and care-related factors play a major role in the incidence of post-ICU psychological distress, research evidence remains inconclusive, particularly around family satisfaction with care, family-related factors such as type of relationship or demographic variables, and patient-related characteristics such as the cause of admission or length of stay.

Given the high prevalence of long-term consequences of critical illness for families, it is important to identify those who are most at risk and to reduce factors that promote psychological distress in family members. We therefore investigated the relationship between family-, patient-, and care-related risk factors for adverse family responses to critical illness and family member depression, anxiety, and posttraumatic stress within the first 3 months post-ICU in a Swiss sample of families of adults treated in ICU.

## Methods

### Aim

The aim was to determine factors that influence post-ICU psychological distress in family members. We hypothesized that family members with higher levels of satisfaction with ICU care would have lower levels of psychological distress following their close others’ ICU stay. We further hypothesized that family members who were female, lived further away, had lower education levels, were spouses / partners, co-habited, and whose critically ill family member was of a younger age and stayed longer or died in ICU would exhibit higher levels of post-ICU psychological distress.

### Design

We conducted a prospective, single-centre observational study. Data were collected from March 2018 to July 2019 using a written questionnaire with several well-validated self-report measures and by extracting data from clinical records.

### Setting, participants, and procedures

The study took place in one surgical-transplant ICU in a 900-bed Swiss University Hospital. Participants were family members of adult persons cared for in ICU for 24 h or longer. Family members were defined as close others from the patient’s perspective or as noted in the clinical record. They had to be 18 years of age and able to fill in the German language questionnaire. No other exclusion criteria were identified.

We used a consecutive sampling strategy. Potential participants were identified from the clinical record. Family members meeting the inclusion criteria received the study information pack together with a written questionnaire by mail after their close other had been discharged from ICU. After a first phase of data collection (March – September 2018) a nurse-led family support service was introduced on the unit. Subsequently, only those family members who received the service in addition to meeting the inclusion criteria were invited to take part (October 2019 – July 2019). Return of the completed written or online questionnaire (https://www.project-redcap.org/) was taken as informed consent. A research assistant followed family members up or sent out written reminders after four and 8 weeks if necessary.

### Measures

#### Independent variables

To assess families’ satisfaction with intensive care, the validated, 24-item German version of the *Family Satisfaction in the ICU (FS-ICU-24)* was used [25, 26]. The FS-ICU-24 assesses on a 5-point-Likert scale the satisfaction with care and with involvement in decision-making. A standardized score of 0–100 is calculated, whereas 100 indicates maximal satisfaction. The FS-ICU-24 has excellent psychometric properties [27], with a high internal consistency (Cronbach’s alpha of > 0.90) [25, 26], also in our sample.

A brief demographic form was used to obtain *patient- and family member-related characteristics*, including family members’ age, gender, educational level, living situation, type of relationship to patient, and distance to hospital. Questions about patient age, gender, and reason of ICU admission were also included in the demographic form. Information on length of stay and death were extracted from the clinical record.

#### Dependent variables

In accordance with definitions of PICS-F [13], we assessed depression, anxiety, and posttraumatic stress. We used the recommended [11, 12, 14] and well-validated German version of the *Hospital Anxiety and Depression Scale (HADS)* [28, 29]. The HADS rates on a 4-point Likert scale anxiety (HADS-A, 7 item, score 0–21) and depression (HADS-D, 7 items, score 0–21), where higher scores indicate worse symptoms. A value of eight or higher has been reported to be indicative of mild depression or anxiety [29, 30], and one of 10 or higher of severe depression or anxiety [11–13]. Cronbach’s alpha of the German version is above 0.80 for both subscales [28]. In our sample, it was 0.86 and 0.88.

To measure stress, we used the 6-item short form of the German version of the *Impact of Event Scale-Revised (IES-R-6)* [31, 32]. The IES-R-6 measures severity of subjective stress on a 5-point Likert scale (score from 0 to 24), with a high score indicating posttraumatic stress. The IES-R-6 is a valid measure (Cronbach’s alpha = 0.80) that correlates highly with the 22-item IES-R. Cronbach’s alpha in our sample was satisfactory with 0.75. A value of over 30 is used in the IES-R-22 item version whose score ranges from 0 to 88, which corresponds with a cut-off value of nine with the shorter six-item version used in our study.

### Data analysis

Data analysis was performed using R version 3.6.1 [33]. To assess the pairwise associations between psychological distress outcomes and continuous independent variables family satisfaction, patient age and family member age, we computed a Pearson correlation matrix between these variables, ignoring the clustering of subjects within families. The outcomes depression, anxiety (HADS subscales) and posttraumatic stress (IES-R-6) were analyzed by linear mixed-effects models with a random intercept per patient to account for the dependence of family members from the same patient. Family satisfaction with care (FS-ICU-24), the family member characteristics of age, gender, relationship type (spouse / partner vs. other), co-habitation (yes vs. no), educational level (tertiary / university vs. other), distance to hospital (same region vs. other) and the patient characteristics age, gender, cause of ICU admission (unplanned admission is used as reference category), length of ICU stay (> 5 days vs. ≤ 5 days), and death were used as explanatory variables in the model. Because one third of participants (*n* = 75) took part in the study after a new family support service was introduced, receiving the nurse-led family support service (yes vs. no) was also included as an explanatory variable in the model. Time-point of completion of the questionnaire (< 4 weeks vs. < 4 weeks) was also entered into the model to control for the effect of time.

### Ethical approval

The study was reviewed by the Ethics Committee of the Canton of Zurich, which waived the need for approval (Req-2018-00107). We followed national guidelines of Research with Humans [34].

## Results

### Patient and family member characteristics

**Fig. 1:**
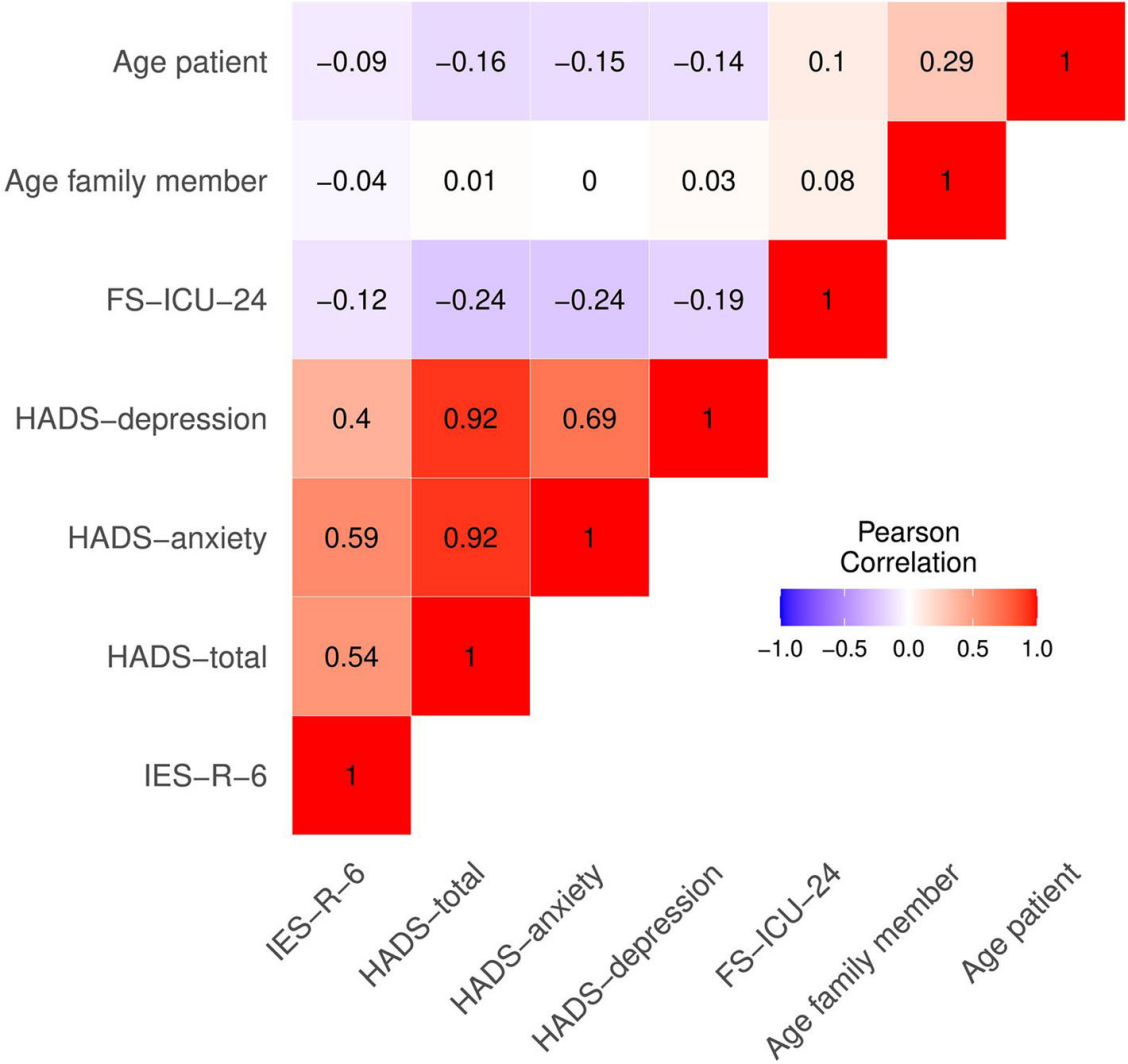
Heatmap of Person correlation coefficients for outcome variables and continuous explanatory variables

A total of 214 family members from 193 patients participated in the study (response rate of 53%). Fifty percent returned a questionnaire within 4 weeks after their patient discharge or death. Sample characteristics are listed in Table 1 and patient variables in Table 2. About one fifth (22.4%, *n* = 47) of family members reported mild or severe levels of depression (HADS-depression subscale > 8) and over one third (38.9%, *n* = 82) mild or severe levels of anxiety (HADS-anxiety subscale > 8). Posttraumatic stress symptoms (IES-R-6 score > 9) were evident in two thirds (67.6%, *n* = 144) of family members. Psychological distress outcomes were positively correlated among themselves, but were negatively correlated with family satisfaction with care and patient and family member age (Fig. 1).

**Table 1:** Family member characteristics and outcome measures

**Family members characteristics**	***n*** **= 214**	**Missing values**
**Age**^**a**^ mean ± SD	53.95 ± 15.67	5
**Female gender**^**a**^ n(%)	148 (69.2)	–
**Civil status** n(%)		1
married, partnered	154 (72.3)	
divorced, separated	12 (5.6)	
single	35 (16.4)	
widowed	12 (5.6)	
**Own children, yes** n(%)	150 (71.1)	3
**Education**^**a**^ n(%)		2
Non-tertiary	109 (51.4)	
Tertiary or university	103 (48.6)	
**Employment** n(%)		1
employed	115 (54.0)	
retired	65 (30.5)	
unemployed	10 (4.7)	
other	23 (10.8)	
**Distance home-to-hospital**^**a**^ n(%)		–
Same canton / region	109 (50.9)	
Outside canton / region	105 (49.1)	
**Relation of family member to patient**^**a**^ n(%)		–
spouse / partner	97 (45.3)	
child	57 (26.6)	
parent	28 (13.1)	
other family member	32 (15.0)	
**Co-habiting with patient before admission**^**a**^ n(%)	119 (55.6)	–
**Time since ICU discharge > 4 weeks**^**a**^ n(%)	107 (50.0)	–
**Satisfaction with ICU care**^**a**^ **(FS-ICU-24**^**b**^**)** mean ± SD	73.54 ± 16.26	5
**Family members outcomes**	***n =*** **214**	**Missing values**
**Psychological burden (HADS-total**^**c**^**)** mean ± SD	11.49 ± 8.12	5
**Depression (HADS-depression**^**c**^**)** mean ± SD	4.69 ± 4.39	5
**Anxiety (HADS-anxiety**^**c**^**)** mean ± SD	6.81 ± 4.42	3
**Posttraumatic stress (IES-R-6**^**d**^**)** mean ± SD	11.90 ± 4.90	2

^a^included in the model as an explanatory variable

^b^FS-ICU-24 score ranges from 0 to 100 (maximal satisfaction)

^c^HADS-total score ranges from 0 to 42 (high psychological burden), HADS-depression and HADS anxiety from 0 to 21 (high depressive symptoms / anxiety)

^d^IES-R-6 score from 0 to 24 (posttraumatic stress)

**Table 2:** Patient characteristics

	***n*** = 193
**Age**^**a**^ mean ± SD	60.84 ± 16.77
**Female gender**^**a**^ n(%)	82 (42.5)
**Civil status** n(%)	
married, partnered	123 (63.7)
divorced, separated	25 (13.0)
single	32 (16.6)
widowed	13 (6.7)
**Living situation** n(%)	
alone	50 (25.9)
with > one family member	137 (71.0)
with someone else	6 (3.1)
**Cause of admission**^**a**^ n(%)	
unplanned	77 (39.9)
transfer from other ICU	20 (10.4)
organ transplant	38 (19.7)
elective surgery	58 (30.1)
**Length of ICU stay**^**a**^ median [IQR]	5 [2,12]
**Died in ICU**^**a**^**, yes** n(%)	38 (19.7)

^a^included in the model as an explanatory variable

There were no missing values in patient variables

### Depression

Depression (HADS-depression) was negatively associated with family satisfaction with intensive care (FS-ICU-24 estimated regression coefficient of − 0.064, 95% confidence interval from − 0.11 to − 0.02, *p* = 0.01, Table 3), suggesting that higher levels of satisfaction correspond with lower levels of depression following a close other’s ICU stay. Higher patient age was associated with lower family member depression levels (coefficient of − 0.052, 95% CI − 0.10 to − 0.00, *p* = 0.038) whereas patient death was associated with higher family member depression levels (coefficient of 3.186, 95% CI 1.54 to 4.83, *p* = 0.0002). Moreover, organ transplantation as cause of admission was associated with the lowest depressive symptoms compared to other causes of admission, the reduction compared to unplanned admission was estimated as − 1.832 (95% CI − 3.79 to 0.12, *p* = 0.062). However, this association did not show - like all other patient or family member characteristic - a statistically significant association with depression.

**Table 3:** Associations between explanatory variables and depression, anxiety, and posttraumatic stress

	Depression (HADS-depression)^**a**^			Anxiety (HADS-anxiety)^**a**^			Posttraumatic stress (IED-R-6)^**a**^		
Model term	Estimate	95% CI^**b**^	***p***-Value	Estimate	95% CI^**b**^	***p***-Value	Estimate	95% CI^**b**^	***p***-Value
**(Intercept)**	4.736	from 2.57 to 6.90	< 0.0001	7.095	from 4.89 to 9.31	< 0.0001	12.848	from 10.41 to 15.29	< 0.0001
**Satisfaction with care (FS-ICU-24)**	−0.064	from −0.11 to − 0.02	0.01	− 0.063	from − 0.11 to − 0.02	0.01	− 0.054	from − 0.10 to − 0.00	0.037
**Age of family member**	0.024	from − 0.03 to 0.08	0.31	0.020	from − 0.03 to 0.07	0.40	0.015	from − 0.04 to 0.07	0.57
**Female gender of family member**	−0.677	from −2.38 to 1.03	0.38	0.546	from −1.13 to 2.22	0.47	0.436	from − 1.53 to 2.40	0.62
**Being the spouse or partner**	0.058	from −2.38 to 2.49	0.96	0.335	from −2.13 to 2.80	0.76	−1.475	from −4.27 to 1.32	0.25
**Co-habiting with critically ill person**	1.533	from −0.77 to 3.83	0.16	0.152	from −2.19 to 2.50	0.88	1.276	from −1.33 to 3.88	0.29
**Higher education level**	−0.183	from −1.60 to 1.24	0.77	−0.292	from −1.76 to 1.18	0.66	−0.799	from −2.40 to 0.80	0.28
**Living closer to hospital**	0.029	from −1.41 to 1.47	0.96	−0.457	from −1.96 to 1.04	0.50	−0.651	from −2.25 to 0.95	0.37
**Family support service, yes**	0.880	from −0.62 to 2.38	0.25	0.346	from −1.28 to 1.97	0.68	0.337	from −1.33 to 2.00	0.69
**Time since ICU discharge > 4 weeks**	−0.895	from −2.30 to 0.50	0.17	−0.574	from −1.99 to 0.84	0.38	−0.645	from −2.23 to 0.94	0.37
**Age of patient**	−0.052	from −0.10 to − 0.00	0.038	−0.048	from − 0.10 to 0.00	0.059	− 0.021	from − 0.08 to 0.03	0.39
**Female gender of patient**	−0.743	from −2.15 to 0.66	0.30	−0.011	from −1.50 to 1.48	0.99	−0.682	from −2.25 to 0.88	0.39
**Cause of ADM: transfer from other ICU**^c^	−0.746	from −3.11 to 1.62	0.48	−0.888	from −3.34 to 1.56	0.43	−1.062	from − 3.71 to 1.59	0.37
**Cause of ADM: organ transplant**^c^	−1.832	from −3.79 to 0.12	0.062	−1.407	from −3.42 to 0.60	0.15	−0.913	from −3.12 to 1.29	0.36
**Cause of ADM: elective surgery**^c^	0.247	from −1.54 to 2.03	0.75	0.348	from −1.48 to 2.18	0.67	−1.036	from −3.04 to 0.97	0.26
**Length of ICU stay** **>** **5 days**	−0.655	from −1.99 to 0.68	0.33	−0.821	from −2.25 to 0.61	0.26	−0.080	from −1.57 to 1.41	0.92
**Death of patient in ICU, yes**	3.186	from 1.54 to 4.83	0.0002	1.146	from −0.64 to 2.93	0.21	3.096	from 1.31 to 4.88	0.0008

^a^Coefficient estimates from a linear mixed effects model on the HADS-depression and HADS-anxiety of family members from all patients. A random intercept was modelled per patient. A total of 198 family members from 179 patients were included in the HADS-depression models. A total of 200 family members from 180 patients were included in the HADS-anxiety model. A total of 201 family members from 182 patients were included in the IES-R-6 posttraumatic stress model

^b^confidence interval

^c^reference category is unplanned admission to ICU

### Anxiety

A similar pattern was evident in associations of the explanatory variables with the HADS-anxiety subscale. Anxiety was negatively associated with family satisfaction with care (estimated regression coefficient of − 0.063, 95% CI − 0.11 to − 0.02, *p* = 0.01) and patient age (coefficient of − 0.048, 95% CI − 0.10 to 0.00, *p* = 0.059), although the latter was statistically non-significant. There was also no significant association with cause of admission and patient death (Table 3). None of the other patient and family member characteristics were statistically significantly associated with anxiety.

### Posttraumatic stress

Posttraumatic stress also showed a negative association with satisfaction with care (estimated regression coefficient of − 0.054, 95% CI from − 0.10 to − 0.00, *p* = 0.037) and a positive association with patient death (coefficient of 3.096, 95% CI from 1.31 to 4.88, *p* = 0.0008). No other explanatory variables were statistically significant (Table 3).

## Discussion

This cross-sectional study with 214 family members of adult persons treated in ICU examined the relationships of care-, family member-, and patient-related risk factors with depression, anxiety and posttraumatic stress. We found that higher levels of family satisfaction with ICU care were associated with lover levels of all three measures of psychological distress (depression, anxiety and posttraumatic stress). While family member characteristics were not significantly associated with any measure of psychological distress, higher patient age was associated with lover levels of depression, and less conclusively with anxiety. Patient death was associated with higher levels of depression and posttraumatic stress.

Our study confirms a high prevalence of post-intensive care syndrome in family members of critically ill persons within the first weeks post-ICU discharge or death. Posttraumatic stress was more prevalent in our sample than previously reported, whereas anxiety and depression occurred in a similar proportion of family members compared to other studies [9, 35–37]. The higher rate of posttraumatic stress might be due to the fact that data were collected relatively shortly after ICU discharge or death, when the ramifications of critical illness are still acute [1, 19, 20]. Moreover, different cut-off levels have been used for the HADS and IES-R, which makes comparisons across studies more difficult [10, 13].

Satisfaction levels in our sample were high, but within the ranges reported in other studies [38, 39]. Our hypothesis about the relationship between family satisfaction with intensive care and psychological distress was confirmed. Those family members who were more satisfied with the quality of care also experienced lower levels of depression, anxiety, and posttraumatic stress. Based on our data set, the direction of the association remains unclear and cannot be assessed because satisfaction and psychological distress variables were measured at the same time. A previous study, however, suggested that lower satisfaction with information and decision-making predicts depression, but not anxiety [40]. The consistency across all three outcomes is notable, as it suggests that quality of patient and family care might be closely associated with the negative consequences of ICU treatment on family members’ health and well-being. Previous research has found that insufficient communication and lack of involvement in decision-making increases level of post-ICU anxiety and posttraumatic stress [9, 19, 24]. Satisfaction is higher in those families whose needs have been met, particularly in relation to communication and decision-making preferences [41–43]. Another study reported that nurse competency, concern and caring, completeness of information, and dissatisfaction with decision-making were independent predictors of dissatisfaction with ICU care [44]. Hence, a focus on amendable factors of ICU care is necessary. It seems that investing in family support and quality of communication may improve satisfaction with ICU care, and therefore be potentially relevant for reducing negative ramifications for family member post-ICU mental health [45, 46]. However, our study is unable to make inferences around the causal direction of the relationship among satisfaction with intensive care and post-ICU psychological distress. Moreover, the relationship between patient outcomes and family satisfaction remains unclear. While some found that satisfaction with care is higher in family members of patients who died than in those who survived [47], others did not identify such a difference [38, 44].

Our study did not identify a statistically significant relationship between family members’ characteristics and psychological distress measures. Previous research has identified that female gender, younger age, and lower education levels are associated with depression, and to a lesser extent with anxiety and stress at some point within the first 6 months post-ICU [3, 9, 18–22, 37, 48]. Being a spouse or an adult child has also been found to be associated with higher levels of psychological distress [1, 8, 19, 37]. However, no clear pattern is evident in terms of the exact outcome predicted, and findings vary among studies. Our study suggests that overall, pre-existing family-related characteristics, such as age, gender and type of relationship may play a subordinate role among predictor variables for family member post-ICU psychological distress. While these pre-ICU family member characteristics have come to be commonly understood as risk factors [10, 11, 13, 15], their evidence base might be less clear than suggested [22, 23].

Among the investigated patient-related factors, younger patient age was negatively associated with depression and anxiety, although not to a statistically significant extent when it comes to anxiety. It seems intuitive that critical illness in younger persons might be less expected, therefore increasing depression post-ICU. In fact, some studies found that younger age is associated with depression, anxiety, or posttraumatic stress in family members [23, 49]. However, other research was unable to find a statistically significant relationship between patient age and family members’ psychological outcomes [20]. Patient death was associated with higher levels of depression and posttraumatic stress, but not with anxiety in our study. It may well be that anxiety is less in the foreground after the close other has died, as symptoms of depression and posttraumatic stress move more to the foreground. It is not surprising that bereaved family members experience psychological distress, particularly so when end-of-life and circumstances of dying were difficult for family members and associated with uncertainty and helplessness [9, 37].

The non-significant, but notable negative relationship between organ transplantation and depression levels might be due to the fact that organ transplantation instills hope, as it enables a second chance for life. As such, families might be less depressed following an ICU stay for transplantation, but still experience anxiety and stress alike family members whose close others were admitted to ICU for other reasons. This might also explain why other causes of admission were not statistically significantly associated with adverse health outcomes, even though unexpected illness or unplanned admission have been identified as a risk factor for family member psychological distress [11, 13]. Some have also found that indicators of severity of illness, such as the presence of mechanical ventilation, a more dynamic illness course or longer length of stays in ICU impact negatively on family member mental health [8, 21]. We only assessed length of ICU stay and found no association with family member psychological distress, along with others [22, 50].

### Limitations

Our study is not without limitations. First, it denotes a secondary analysis of a single-center study [51]. Only half of those invited to take part did return a completed questionnaire. As a consequence, representativeness of our sample of the population remains unclear, and generalizability of our findings remains limited. The care offered to families changed during the course of the study, with a family nurse delivered support service that was introduced to the unit [52]. While we derived our hypothesis from pre-existing research, we were not able to assess all potential risk factors, such as severity of illness, duration of relationship, quality of communication or end-of-life care, which limits our models. We had only one time-point of assessment, which varied between one to twelve weeks post-ICU discharge or death, with about half returning a questionnaire within the first 4 weeks. Moreover, satisfaction with ICU care was measured at the same time as psychological distress, making inferences about causal relationships impossible. These design limitations need to be taken into account when interpreting our study’s results. Nonetheless, this study uses a solid data set with a sufficient number of participants. It utilized well-established and psychometrically sound instruments to assess family outcomes. As such, it adds important research-based insights into pre- and intra-ICU risk factors for post-ICU psychological distress in families.

## Conclusions

Our findings indicate that family members of younger patients and of those who die are at risk for adverse mental health outcomes, and may need specific support, particularly at the end-of-life and into bereavement. Family-related factors, however, may play a subordinate role. The potentially amendable, care-related factor - families’ satisfaction with ICU care – was strongly, and most clearly, associated with family well-being post-ICU. ICU staff is therefore called to ensure high-quality ICU care to families that is tailored to their needs and preferences, consisting of optimal information provision, ongoing communication and interaction, and emotional and practical support. ICU policies around family involvement, engagement and support need to guarantee sufficient staff capacity and skills in working with families. Future research is needed to discern the pathways and the direction of association between satisfaction with care and similar, amendable indicators of quality of ICU care with family member psychological distress. Multi-centre studies with larger, more representative and homogenous samples that assess psychological distress longitudinally at fixed time intervals will increase understanding of factors predicting family members’ psychological distress post-ICU.

## Data Availability

The datasets used and/or analysed during the current study are available from the corresponding author on reasonable request.

## Abbreviations

ICU: Intensive Care Unit
PICS-F: Post-Intensive Care syndrome – Family
FS-ICU-24: Family Satisfaction with Intensive Care Survey
HADS: Hospital Anxiety and Depression Scale
IES-R-6: Impact of Event Scale - Revised short version

## References

[1] Alfheim HB, Hofso K, Smastuen MC, Toien K, Rosseland LA, Rustoen T (2019). Post-traumatic stress symptoms in family caregivers of intensive care unit patients: a longitudinal study. Intensive Critical Care Nurs.

[2] Alfheim HB, Smastuen MC, Hofso K, Toien K, Rosseland LA, Rustoen T (2018). Quality of life in family caregivers of patients in the intensive care unit: a longitudinal study. Aust Critical Care.

[3] Anderson WG, Arnold RM, Angus DC, Bryce CL (2008). Posttraumatic stress and complicated grief in family members of patients in the intensive care unit. J Gen Intern Med.

[4] Minton C, Batten L, Huntington A (2019). A multicase study of prolonged critical illness in the intensive care unit: families’ experiences. Intensive Critical Care Nurs.

[5] Turner-Cobb JM, Smith PC, Ramchandani P, Begen FM, Padkin A (2016). The acute psychobiological impact of the intensive care experience on relatives. Psychol Health Med.

[6] Hopkins RO, Netzer G (2018). Emotional processing / pschological morbidity in the icu. Families in the intensive care unit: a guide to unerstanding, engaging, and supporting at the bedside.

[7] Harvey MA, Davidson J (2011). Long-term consequences of critical illness: a new opportunity for high-impact critical care nurses. Crit Care Nurse.

[8] Siegel MD, Hayes E, Vanderwerker LC, Loseth DB, Prigerson HG, Siegel MD (2008). Psychiatric illness in the next of kin of patients who die in the intensive care unit. Crit Care Med.

[9] Azoulay E, Pochard F, Kentish-Barnes N, Chevret S, Aboab J, Adrie C (2005). Risk of post-traumatic stress symptoms in family members of intensive care unit patients. Am J Respir Crit Care Med.

[10] Inoue S, Hatakeyama J, Kondo Y, Hifumi T, Sakuramoto H, Kawasaki T (2019). Post-intensive care syndrome: its pathophysiology, prevention, and future directions. Acute Med Surg.

[11] McAdam JL, Puntillo K (2009). Symptoms experienced by family members of patients in intensive care units. Am J Crit Care.

[12] Kentish-Barnes N, Lemiale V, Chaize M, Pochard F, Azoulay E (2009). Assessing burden in families of critical care patients. Crit Care Med.

[13] Davidson JE, Jones C, Bienvenu OJ (2012). Family responses to critical illness: Postintensive care syndrom - family. Crit Care Med.

[14] van Beusekom I, Bakhshi-Raiez F, de Keizer NF, Dongelmans DA, van der Schaaf M (2016). Reported burden on informal caregivers of icu survivors: a literature review. Crit Care.

[15] Serrano P, Kheir YNP, Wang S, Khan S, Scheunemann L, Khan B (2019). Aging and postintensive care syndrome-family: a critical need for geriatric psychiatry. Am J Geriatr Psychiatry.

[16] Torres J, Veiga C, Pinto F, Ferreira A, Sousa F, Jacinto R (2015). Caregiving burden: the impact of post intensive care syndrome. Intensive Care Med Exp.

[17] Wintermann GB, Petrowski K, Weidner K, Strauß B, Rosendahl J (2019). Impact of post-traumatic stress symptoms on the health-related quality of life in a cohort study with chronically critically ill patients and their partners: age matters. Crit Care.

[18] Haines KJ, Denehy L, Skinner EH, Warrillow S, Berney S (2015). Psychosocial outcomes in informal caregivers of the critically ill: a systematic review. Crit Care Med.

[19] Gries CJ, Engelberg RA, Kross EK, Zatzick D, Nielsen EL, Downey L (2010). Predictors of symptoms of posttraumatic stress and depression in family members after patient death in the ICU. Chest..

[20] Cameron JI, Chu LM, Matte A, Tomlinson G, Chan L, Thomas C (2016). One-year outcomes in caregivers of critically ill patients. N Engl J Med.

[21] Chui WY-Y, Chan SW-C (2007). Stress and coping of Hong Kong chinese family members during a critical illness. J Clin Nurs.

[22] Lee RY, Engelberg RA, Curtis JR, Hough CL, Kross EK (2019). Novel risk factors for posttraumatic stress disorder symptoms in family members of acute respiratory distress syndrome survivors. Crit Care Med.

[23] Kross EK, Engelberg RA, Gries CJ, Nielsen EL, Zatzick D, Curtis JR (2011). Icu care associated with symptoms of depression and posttraumatic stress disorder among family members of patients who die in the ICU. Chest..

[24] Rusinova K, Kukal J, Simek J, Cerny V (2014). Limited family members/staff communication in intensive care units in the czech and slovak republics considerably increases anxiety in patients' relatives--the depress study. BMC Psychiatry.

[25] Harrison DA, Ferrando-Vivas P, Wright SE, McColl E, Rowan KM, Investigators FS (2015). Psychometric assessment of the family satisfaction in the intensive care unit (fs-icu-24) questionnaire among family members of patients admitted to adult general icus in the United Kingdom. Intensive Care Med Exp.

[26] Stricker KH, Niemann S, Bugnon S, Wurz J, Rohrer O, Rothen HU (2007). Family satisfaction in the intensive care unit: cross-cultural adaptation of a questionnaire. J Crit Care.

[27] van den Broek JM, Brunsveld-Reinders AH, Zedlitz AM, Girbes AR, de Jonge E, Arbous MS (2015). Questionnaires on family satisfaction in the adult icu: a systematic review including psychometric properties. Crit Care Med.

[28] Herrmann-Lingen C, Buss U (2011). P. SR. hospital anxiety and depression scale – deutsche version (HADS-D). Manual.

[29] Bjelland I, Dahl AA, Haug TT, Neckelmann D (2002). The validity of the hospital anxiety and depression scale. An updated literature review. J Psychosom Res.

[30] Lewis CL, Taylor JZ (2017). Impact of prior icu experience on icu patient family members’ psychological distress: a descriptive study. Intensive Critical Care Nurs.

[31] Maercker A, Schützwohl M (1998). assessment of post-traumatic stress reactions: The impact of event scale-revised (ies-r). Diagnostica.

[32] Thoresen S, Tambs K, Hussain A, Heir T, Johansen VA, Bisson JI (2010). Brief measure of posttraumatic stress reactions: impact of event Scale-6. Soc Psychiatry Epidemiol.

[33] Team RC (2020). R: a language and environment for statistical computing.

[34] Swiss Academy of Medical Sciences (2015). Research with human subjects Bern: Swiss Academy of Medical Sciences.

[35] Petrinec AB, Martin BR (2018). Post-intensive care syndrome symptoms and health-related quality of life in family decision-makers of critically ill patients. Palliat Support Care.

[36] Lautrette A, Darmon M, Megarbane B, Joly LM, Chevret S, Adrie C (2007). A communication strategy and brochure for relatives of patients dying in the icu. N Engl J Med.

[37] Matt B, Schwarzkopf D, Reinhart K, König C, Hartog CS (2017). Relatives' perception of stressors and psychological outcomes - results from a survey study. J Crit Care.

[38] Schwarzkopf D, Behrend S, Skupin H, Westermann I, Riedemann NC, Pfeifer R (2013). Family satisfaction in the intensive care unit: a quantitative and qualitative analysis. Intensive Care Med.

[39] Padilla Fortunatti C, Rojas SN (2018). Families on adult intensive care units: are they really satisfied? A literature review. Aust Critical Care.

[40] Metzger K, Gamp M, Tondorf T, Hochstrasser S, Becker C, Luescher T (2019). Depression and anxiety in relatives of out-of-hospital cardiac arrest patients: results of a prospective observational study. J Crit Care.

[41] Khalaila R (2013). Patients' family satisfaction with needs met at the medical intensive care unit. J Adv Nurs.

[42] Scott P, Thomson P, Shepherd A (2019). Families of patients in ICU: a scoping review of their needs and satisfaction with care. Nurs Open.

[43] Bailey JJ, Sabbagh M, Loiselle CG, Boileau J, McVey L (2010). Supporting families in the icu: a descriptive correlational study of informational support, anxiety, and satisfaction with care. Intensive Critical Care Nurs.

[44] Hunziker S, McHugh W, Sarnoff-Lee B, Cannistraro S, Ngo L, Marcantonio E (2012). Predictors and correlates of dissatisfaction with intensive care. Crit Care Med.

[45] Davidson JE, Aslakson RA, Long AC, Puntillo KA, Kross EK, Hart J (2017). Guidelines for family-centered care in the neonatal, pediatric, and adult ICU. Crit Care Med.

[46] Mitchell ML, Coyer F, Kean S, Stone R, Murfield J, Dwan T (2016). Patient, family-centred care interventions within the adult icu setting: an integrative review. Aust Critical Care.

[47] Wall RJ, Curtis JR, Cooke CR, Engelberg RA (2007). Family satisfaction in the icu: differences between families of survivors and nonsurvivors. Chest..

[48] Paparrigopoulos T, Melissaki A, Efthymiou A, Tsekou H, Vadala C, Kribeni G (2006). Short-term psychological impact on family members of intensive care unit patients. J Psychosom Res.

[49] Köse I, Zincircioğlu Ç, Öztürk YK, Çakmak M, Güldoğan EA, Demir HF (2016). Factors affecting anxiety and depression symptoms in relatives of intensive care unit patients. J Intensive Care Med.

[50] Hwang DY, Yagoda D, Perrey HM, Currier PF, Tehan TM, Guanci M (2014). Anxiety and depression symptoms among families of adult intensive care unit survivors immediately following brief length of stay. J Crit Care.

[51] Naef R, von Felten S, Petry H, Ernst J, Massarotto P. Impact of a nurse led - family support intervention on family members’ satisfaction with intensive care and psychological well-being. Aust Critical Care. 2021. in press. 10.1016/j.aucc.2020.10.014 33637427

[52] Naef R, Massarotto P, Petry H (2020). Families’ and health professionals’ experience with a nurse-led family support intervention in icu: a qualitative evaluation study. Intensive Critical Care Nurs.

